# Prize Question of the Lyceum Medicum Londinense

**Published:** 1799-06

**Authors:** John Crake

**Affiliations:** Register


### *To the Editors of the Medical and Phyſical Journal.*

GENTLEMEN,

I Am deſired by the Committee of the Lyceum Medicum Londinenſe, to ſend you the encloſed, being the Prize Queſtion of that ſociety for the enſuing year, and to requeſt you will have the goodneſs to inſert it in your next Number.

I am, Gentlemen,

Your humble Servant,

*Thurſday, May* 23, 1799. JOHN CRAKE, Regiſter.

---

### PRIZE QUESTION.

An acid precipitates a ſubſtance from the bile ſimilar to the reſinous ſubſtances of plants, which is inſoluble in water :—why does this precipitation take place? The ſame ſubſtance is often ſeparated in the gall-bladder and duct, forming concretions:—how is this precipitation produced, and is there any method by which it may be prevented, or by which it, when precipitated, may be re-diſſolved?

